# Maintenance of Basal Levels of Autophagy in Huntington’s Disease Mouse Models Displaying Metabolic Dysfunction

**DOI:** 10.1371/journal.pone.0083050

**Published:** 2013-12-20

**Authors:** Barbara Baldo, Rana Soylu, Åsa Petersén

**Affiliations:** Translational Neuroendocrine Research Unit, Department of Experimental Medical Science, Lund University, Lund, Sweden; University of Florida, United States of America

## Abstract

Huntington’s disease (HD) is a fatal neurodegenerative disorder caused by an expanded polyglutamine repeat in the huntingtin protein. Neuropathology in the basal ganglia and in the cerebral cortex has been linked to the motor and cognitive symptoms whereas recent work has suggested that the hypothalamus might be involved in the metabolic dysfunction. Several mouse models of HD that display metabolic dysfunction have hypothalamic pathology, and expression of mutant huntingtin in the hypothalamus has been causally linked to the development of metabolic dysfunction in mice. Although the pathogenic mechanisms by which mutant huntingtin exerts its toxic functions in the HD brain are not fully known, several studies have implicated a role for the lysososomal degradation pathway of autophagy. Interestingly, changes in autophagy in the hypothalamus have been associated with the development of metabolic dysfunction in wild-type mice. We hypothesized that expression of mutant huntingtin might lead to changes in the autophagy pathway in the hypothalamus in mice with metabolic dysfunction. We therefore investigated whether there were changes in basal levels of autophagy in a mouse model expressing a fragment of 853 amino acids of mutant huntingtin selectively in the hypothalamus using a recombinant adeno-associate viral vector approach as well as in the transgenic BACHD mice. We performed qRT-PCR and Western blot to investigate the mRNA and protein expression levels of selected autophagy markers. Our results show that basal levels of autophagy are maintained in the hypothalamus despite the presence of metabolic dysfunction in both mouse models. Furthermore, although there were no major changes in autophagy in the striatum and cortex of BACHD mice, we detected modest, but significant differences in levels of some markers in mice at 12 months of age. Taken together, our results indicate that overexpression of mutant huntingtin in mice do not significantly perturb basal levels of autophagy.

## Introduction

Huntington’s disease (HD) is a fatal neurodegenerative disorder caused by the amplification of a polyglutamine stretch at the N-terminus of the huntingtin (htt) protein [1]. The disease has traditionally been characterized by motor symptoms which currently are required for the clinical HD diagnosis together with a positive genetic test [2], [3]. HD motor disturbances occur around mid-age and are predominantly caused by basal ganglia dysfunction, affecting particularly the medium sized spiny neurons in the striatum [3], [4]. It has been widely recognized that HD patients also experience severe cognitive and psychiatric disturbances, which occur years before the onset of the motor impairment [5], [6], [7]. These symptoms significantly affect the life of the patients and their relatives [8]. Other non-motor symptoms and signs such as sleep disturbances, changes in the circadian rhythm, autonomic dysfunction and metabolic dysfunction are also thought to occur early in the disease process [9], [10], [11], [12], [13], [14], [15]. The underlying neurobiological changes of the non-motor aspects of HD have not been extensively studied. Cortico-striatal changes are likely to be important for the cognitive changes but less is known about the other non-motor aspects of the disease. Better understanding of these early disease phenotypes could provide important insight into early pathogenic steps in HD.

Recent studies using both magnetic resonance imaging and positron emission tomography have indicated that changes in the hypothalamus occur before motor onset [16], [17]. The hypothalamus constitutes of a number of interconnected nuclei that regulate important functions such as emotion control, metabolism, sleep and the circadian rhythm [18], [19]. Alterations in emotion and metabolism regulating neuropeptides have been demonstrated in human postmortem hypothalamic tissue from HD patients [20], [21], [22], [23]. Experimental studies in mice have recently established a causal link between expression of mutant htt in the hypothalamus and the development of metabolic dysfunction as well as depressive-like behavior in mice [24], [25]. These studies focused on the BACHD mouse which expresses full length mutant htt as well as recombinant adeno-associated viral (rAAV) vectors of serotype 5 engineered to express the first 853 amino acids of htt with either an expanded polyglutamine of 79Q or a normal form with 18Q [24], [25], [26]. The use of rAAV vectors to selectively express a fragment of mutant htt in the hypothalamus led to severe metabolic dysfunction with a rapid body weight gain as well as insulin and leptin resistance [24]. Deletion of mutant htt specifically in the hypothalamus of the BACHD mice prevented the onset of the metabolic phenotype as well as the depressive-like behavior [24], [25]. These data suggest that expression of mutant htt in the hypothalamus is important for the neurocircuitry regulating both food intake and emotions. The key molecular mechanisms by which mutant htt exerts its toxic function in the hypothalamus however remain to be unraveled.

Macroautophagy (from now on referred as autophagy) is an intracellular lysosomal mediated degradation pathway, which is responsible of the clearance of misfolded proteins, aggregates and organelles [27], [28]. Autophagy is important to maintain cellular homeostasis and also to provide the cell with nutrients in starvation conditions [29], [30]. Interestingly, alterations in autophagy have been suggested to play a role in neurodegeneration and particularly in the pathogenesis of HD [28], [31], [32], [33]. Autophagy is thought to contribute to the degradation of both mutant and wild-type (wt) htt and to the processing of mutant htt fragments [34], [35], [36], [37]. Also, the deletion of the polyglutamine stretch in the mutant htt activates autophagy and ameliorates the HD phenotypes in mice [38]. In a similar way, induction of autophagy using inhibitors of its upstream regulator mammalian target of rapamycin (mTOR) have been shown to reduce both neuropathology and behavioral phenotypes in several disease models, promoting clearance of the mutant protein and its aggregates [39], [40], [41]. Finally, the autophagy mediated clearance of mutant htt has been shown to be promoted by the activation of the insulin receptor substrate 2 (IRS-2), which mediates the insulin cascade [42]. Interestingly, autophagy has also been shown to play a role in the control of metabolism and food intake [43]. Recent studies have shown that modulation of the autophagy pathway in specific hypothalamic neuronal populations leads to the development of metabolic dysfunction such as obesity and leptin resistance [44], [45], [46], [47], [48]. Taken together, the role of autophagy in the regulation of hypothalamic function as well as the link between this pathway and HD, opens up for the possibility that dysregulation of the autophagy pathway in the hypothalamus might be involved in hypothalamic dysfunction in HD and the development of metabolic disturbances. In this study we therefore aimed to characterize the basal levels of autophagy in two mouse models displaying metabolic dysfunction, the BACHD mouse and mice injected with rAAV vectors expressing htt fragments in the hypothalamus [24], [26].

## Materials and Methods

### Animals

Female mice from the FVB/N strain were used for stereotactic injections at 6 to 8 weeks of age (Charles Rivers). The BACHD mice express full-length mutant htt with 97 polyglutamine repeats [26]. BACHD males have been obtained from Jacksons Laboratories (Bar Harbor, Maine, USA) and were crossed with FVB/N females. The genotype of the offspring was determined from tail samples using PCR primers (5′-3′): forward CCGCTCAGGTTCTGCTTTTA and reverse AGGTCGGTGCAGAGGCTCCTC. All mice were housed in groups at a 12 h light/dark cycle with ad libitum access to normal chow diet.

#### Ethics statement

All the experimental procedures were approved by the Regional Ethical Committee in Lund, Sweden (Permit number: M20-11).

### Vector Production and Stereotactical Surgery

We injected young female FVB/N mice (6–8 weeks of age) with rAAV vectors of serotype 5 expressing the first 853 amino acids of either the wt form of htt with 18Q (rAAV-htt853-18Q) or the mutant form of the protein with 79Q (rAAV-htt853-79Q). The production of the vectors was performed as previously described and the titers measured by TaqMan quantitative Polymerase Chain Reaction (PCR) with primers and probe directed towards the inverted terminal repeats (ITRs) of the vector [24]. A total of 14 mice per group were injected with the vectors and 14 uninjected mice were used as control. The rAAV vectors had titers of 3.20E+15 and 2.60E+15 GC/ml for the mutant and wt htt respectively. Stereotactic bilateral hypothalamic injections were performed using a 5µl syringe (Hamilton) inserted in a thin glass capillary (unilaterally injected animals have been used for Figure S1). The injections were performed at the following coordinates: −0.6 mm anterio-posterior (AP) and ±0.6 mm medio-lateral (ML) calculated from the Bregma and −5.2 mm dorso-ventral (DV) from the dura. 0.5 µl of vector were injected for each side at a speed of 0.05µl every 15 sec, followed by 5 min wait before a slow retraction of the capillary.

### Body Weight Measurements

The body weight of rAAV5-htt853 vector injected mice as well as the uninjected controls was monitored from the surgery day every two weeks until 8 weeks post injection.

### Collection of Brain Tissue

The cull of the animals was performed by decapitation after anesthesia with sodium-pentobarbital (Apoteksbolaget). Hypothalami from rAAV5-htt853 vector injected mice and uninjected controls were dissected 8 weeks post-injection from 2 coronal sections obtained with a mouse brain matrix (1 mm thickness). The tissue was freshly frozen in liquid nitrogen for Western blot (n = 7/group) or quantitative Real Time – PCR (qRT-PCR) (n = 7/group). The same procedure was applied for hypothalami, cortex and striata of BACHD mice and gender- and age-matched wt animals (2, 6 and 12 months). For the BACHD mice, tissue from one hemisphere was used for Western blot while the other hemisphere was used for quantitative RT-PCR (n = 3–6/group). The animals were kept in normal chow diet until the cull. For the immunohistochemical analyses, brains from female BACHD mice and wt controls were studied at 12 months of age while brains from mice injected unilaterally with rAAV5-htt853 were studied 6 weeks post injection (See Material and Methods S1).

### qRT-PCR

mRNA expression of several autophagy markers and related pathways was analyzed by qRT-PCR from hypothalami dissected from rAAV5-htt853 vector injected mice and uninjected controls (n = 7/group) as well as hypothalamic, striata and cortex from 2, 6 and 12 months old female BACHD mice and gender- and age-matched wt animals (n = 3–6/group). The expression level of the following autophagy genes was analyzed: microtubule-associated protein 1-light chain 3 alpha (LC3A), microtubule-associated protein 1-light chain 3 beta (LC3B), autophagy-related 5 (Atg5), autophagy-related 7 (Atg7), Bcl-2-interacting myosin like coiled-coil protein (Beclin1), sequestosome 1 (p62), lysosomal-associated membrane protein 2 (LAMP2), mammalian target of rapamycin (mTOR), regulatory-associated protein of mTOR (Raptor) and Rapamycin-insensitive companion of mTOR (Rictor) (Table 1). Total RNA was extracted using the RNeasy Lipid Tissue kit (Qiagen) including a step with DNase digestion (RNase free DNase Set, Qiagen) following the manufacturer instructions. cDNA was generated using random primers and SuperScript III Reverse Transcriptase (Invitrogen) following the manufacturer instructions. qRT-PCR was performed in a LightCycler 480 (Roche) using a two-step cycle protocol using SYBR Green I Master (Roche). Calculations were performed with the ΔΔCT method [49], [50]. The relative expression of the genes was normalized to the expression in the same sample of three housekeeping genes; beta-actin, hypoxanthine-guanine phosphoribosyl-transferase and glyceraldehyde 3-phosphate dehydrogenase. After normalization all values were presented as a ratio to the average of the uninjected controls or the 2 months old wt mice. The primer sequences used for the gene expressions analyses are found in Table S1.

**Table 1:** Autophagy genes investigated by qRT-PCR and Western blot.

Gene	Function	Use (WB/qRT-PCR)	References
LC3B	Involved in autophagosome formation	WB/qRT-PCR	[59], [104], [105]
LC3A	Involved in autophagosome formation	qRT-PCR	[59], [104], [105]
Atg5	Involved in the elongation of the phagophore membrane - Conjugated with Atg12	qRT-PCR	[30], [106]
Atg7	Inducing Atg5–Atg12 conjugation and LC3I to LC3II conversion	WB/qRT-PCR	[29], [30]
Beclin1	Involved in membrane nucleation	WB/qRT-PCR	[61], [63], [107]
p62	Recognition of ubiquitinated proteins for selective autophagy - itself degraded by autophagy	WB/qRT-PCR	[108], [109]
LAMP2	Lysosomal-membrane bound protein	qRT-PCR	[110], [111]
mTOR	Negative regulator of autophagy	qRT-PCR	[112], [113]
Raptor	Subunit of mTORC1	qRT-PCR	[112], [114]
Rictor	Subunit of mTORC2	qRT-PCR	[112], [115]
pS6	Ribosomal protein - downstream target of mTORC1 cascade	WB	[102], [112]

### Western Blot Analysis

Hypothalami from rAAV5-htt853 vector injected mice and uninjected controls were dissected 8 weeks post injection and freshly frozen in liquid nitrogen. The same procedure was applied for hypothalami, cortex and striata of 2, 6 and 12 months old female BACHD mice and gender- and age-matched wt animals (n = 3–6/group). The protein levels of the following autophagy markers were assessed: LC3I/II, Atg7, Beclin1, p62 and pS6 (Table 1). The samples were lysed in 1∶10 weight/volume in lysis buffer (50 mM NaCl, 100 mM Tris-HCl pH 7.4, 1 mM EDTA, 1% SDS) supplemented with protease and phosphatase inhibitors (Roche) and sonicated 2 times for 15 sec at 40 Hz. The samples were then incubated for 15 min on ice and spun at 13.000 rpm for 15 min. The protein concentration was measured using the DC Protein Assay Kit (Bio-Rad) following the instructions of the manufacturer. The protein lysates were boiled at 95°C for 10 min in presence of Laemmli Loading Buffer (Bio-Rad). 40 µg of each sample were loaded on a 4–15% gradient SDS-polyacrylamide gel (Bio-Rad Mini-PROTEAN TGX Precast Gels) and run for 30 min at 90 V, followed by 1 h at 120 V. The proteins were then transferred to a PVDF membrane using the Trans-Blot Turbo Transfer System (Bio-Rad). The membranes were blocked for 1 h at room temperature (RT) in 5% skim-milk or 5% BSA in Tris-buffered saline buffer +0.1% Tween 20 (Sigma) (TBS-T). The membranes were then incubated at 4°C overnight with primary antibodies diluted in 2% skim-milk or BSA in TBS-T. The following primary antibodies were used: 1∶1000 rabbit anti-LC3 (NB100-2220, Novus Biologics), 1∶5000 rabbit anti-Beclin1 (NB110-87318, Novus Biologics), 1∶500 rabbit anti-Atg7 (ab53255, Abcam), 1∶1000 rabbit anti-p62 (#5114, Cell Signaling), 1∶1000 rabbit anti-pS6 (#4656S, Cell Signaling) 1∶2000 mouse anti htt (MAB2166, Millipore) and 1∶10000 mouse anti-Beta-Actin (A1978, Sigma-Aldrich). After three washes of 10 min in TBS-T, the membranes were incubated for 1 h at RT with 1∶10000 peroxidase-labeled secondary antibody goat anti-rabbit (Santa Cruz) or goat anti-mouse (Santa Cruz) diluted in 2% skim-milk in TBS-T. Following three washes of 10 min in TBS-T the immuno-complexes were visualized using the Immun-Star WesternC Kit (Bio-Rad) and the Molecular Imager® VersaDocTM MP 4000 System (Bio-Rad). The densitometry analysis of the bands was performed using the computerized image analysis tool Image Lab version 2.0.1.

### Statistical Analysis

The data are presented as mean ± SEM. Statistical analyses were performed using SPSS 18 statistical program (SPSS Inc. Chicago, Il). Statistical difference was considered at p<0.05. One- way analysis of variance (ANOVA) followed by Bonferroni post hoc test or two-way ANOVA followed by post hoc un-paired Student’s T-test were used when appropriate.

## Results

### Development of Metabolic Dysfunction after Selective Expression of htt Fragments in the Hypothalamus

**Figure 1:**
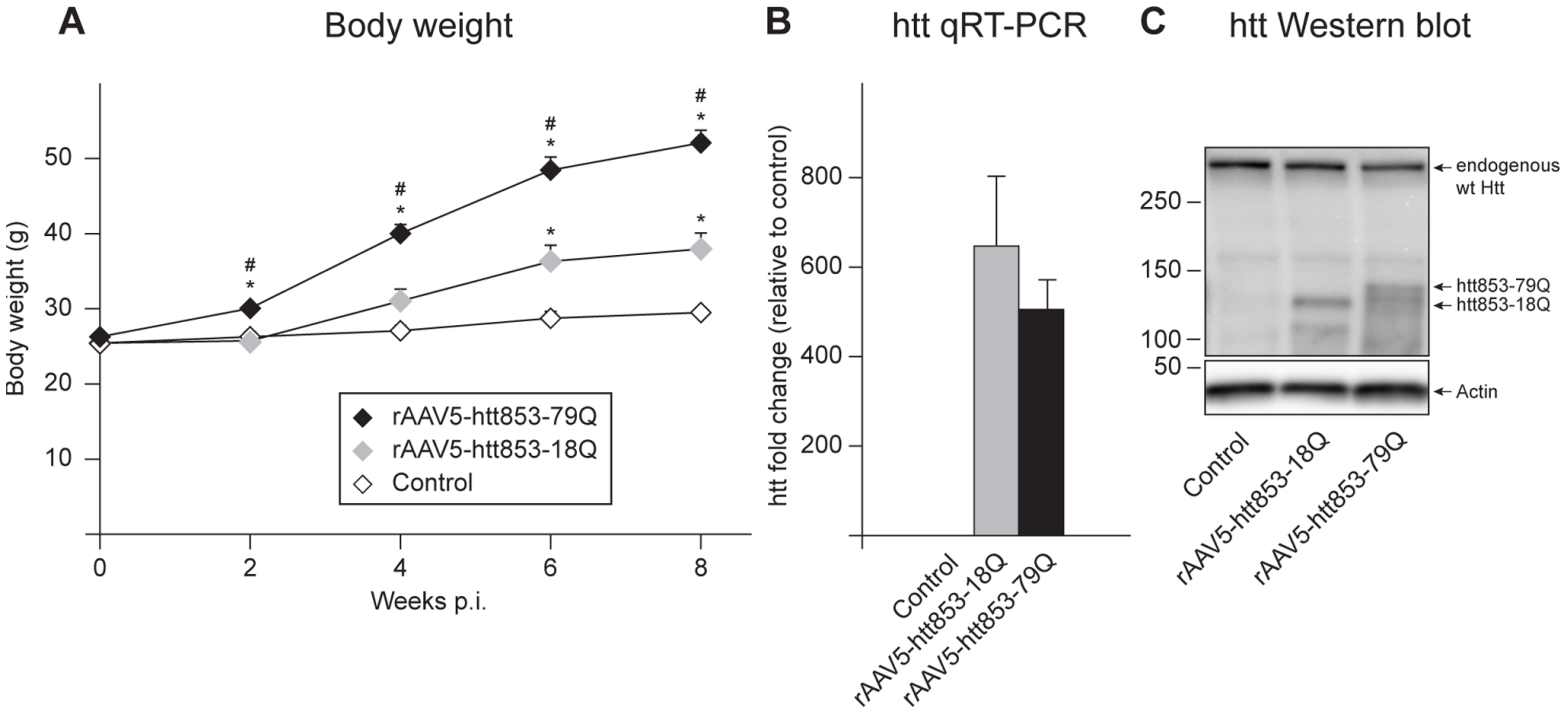
Body weight changes in mice injected with rAAV-htt853 vectors and validation of htt expression. A) Mice bilaterally injected with rAAV5-htt853-79Q displayed increased body weight compared to mice injected with rAAV5-htt853-18Q and uninjected animals already 2 weeks post injection. Animals bilaterally injected with rAAV5-htt853-18Q showed increased body weight compared to uninjected controls 6 weeks post injection (n = 14/group; * and ^#^p<0.05 after Bonferroni post-hoc test; *statistical significant difference compared to uninjected controls while # expresses statistical significance compared to rAAV5-htt853-18Q). B) qRT-PCR verified the expression of htt853-18Q and htt853-79Q relative to uninjected controls in mice 8 weeks post-injection of rAAV5-htt853 vectors (n = 7/group). C) Representative Western blot detected the expression of endogenous wt htt as well as htt853-18Q and htt853-79Q in mice 8 weeks post injection of rAAV5-htt853 vectors.

Female FVB/N mice of 6–8 weeks of age were bilaterally injected in the hypothalamus with rAAV vectors of serotype 5 expressing 853 amino acids of wt or mutant htt, containing 18Q (rAAV5-htt853-18Q) or 79Q (rAAV5-htt853-79Q), respectively. The body weight of rAAV5-htt853-18Q and rAAV5-htt853-79Q mice as well as uninjected controls was monitored every two weeks for 8 weeks post injection (Figure 1A). The successful expression of either wt or mutant htt forms in rAAV5-htt853-18Q and rAAV5-htt853-79Q mice was verified by qRT-PCR as well as Western blot (Figure 1B, C). Similar expression level of mRNA of wt and mutant forms of htt was demonstrated using qRT-PCR (Figure 1B). In accordance to previously published results, the expression of mutant htt lead to a fast and progressive gain of weight [24]. We observed that already 2 weeks post injection the rAAV5-htt853-79Q mice had significantly higher body weight compared to rAAV5-htt853-18Q and to uninjected controls (p<0.05, See Statistical Results S1) (Figure 1A). Interestingly, the presence of wt htt also influenced the body weight of the animals, leading to an increase in body weight significant from uninjected controls from 6 weeks post injection. However, the increase in body weight seen in rAAV5-htt853-79Q mice was significantly higher than the body weight observed in rAAV5-htt853-18Q animals at all the time points (p<0.05, See Statistical Results S1) (Figure 1A). This result suggests that both wt and mutant htt influence metabolic control in the hypothalamus although the effects of mutant htt are stronger.

### Maintenance of Basal Levels of Autophagy in Mice with Selective Expression of htt Fragments in the Hypothalamus

**Figure 2:**
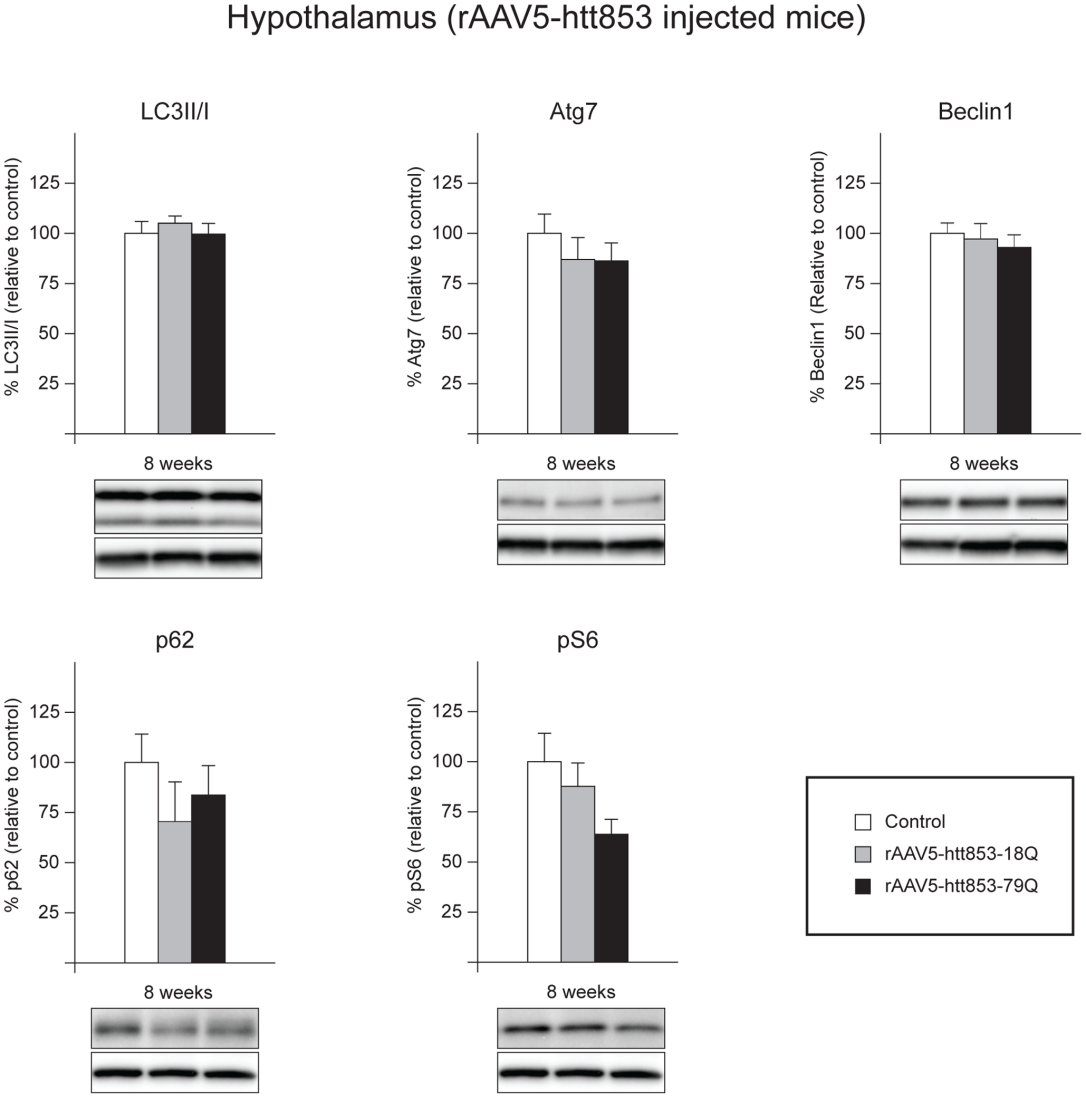
Maintenance of basal levels of autophagy in mice overexpressing htt fragments in the hypothalamus. The expression levels of selected autophagy markers were analyzed by Western blot in mice bilaterally injected with rAAV5-htt853-18Q or rAAV5-htt853-79Q and compared to uninjected controls 8 weeks post injection (n = 6–7/group). The data displayed maintenance of the autophagy flux, despite the presence of a clear metabolic phenotype in the injected mice. The data in the graphs are expressed as mean ± SEM relative to the uninjected controls. The Western blots are representative of one sample for each group.

The metabolic dysfunction induced by the expression of htt fragments in the hypothalamus suggests that the key regulatory metabolic mechanisms might be altered in this region. As changes in autophagy in the hypothalamus have been found to disrupt metabolic control in the hypothalamic region and this process has been suggested to be involved in HD [28], [43], we were interested in investigating whether the autophagic pathway was affected in our mouse model. To this aim, rAAV5-htt853 vector injected animals, as well as gender- and age-matched uninjected controls were euthanized 8 weeks post injection and the hypothalami were dissected. Since mutant htt has been shown to influence gene transcription in clinical HD as well as in animal models, we decided to explore possible changes in autophagy at the transcriptional level as well as at the protein level [51], [52], [53], [54], [55], [56], [57], [58]. We began by performing qRT-PCR analysis of multiple markers of autophagy as well as related pathways, such as the mTOR pathway (n = 6–7/group). Our data suggest that the presence of wt or mutant htt fragments in the hypothalamus of HD mice did not alter the expression of these autophagy markers (Table 2, see Statistical Results S1). Although no significant differences could be observed in expression levels of different markers of the autophagy pathway between the rAAV5-htt853-79Q mice, rAAV5-htt853-18Q mice or uninjected controls, we observed a trend in reduction in components of mTOR (One-factor ANOVA, p = 0.085) by qRT-PCR analysis. We also analyzed selected autophagy markers, as LC3II/I conversion, Atg7, Beclin1 and p62 by Western blot (n = 6–7/group) [29], [59], [60], [61], [62], [63], [64]. The results did not show any significant alterations in the expression levels of these components, suggesting that the basal levels of autophagic flux were not altered in the hypothalamic region of these mice (Figure 2). Since we observed a trend for a reduction of mTOR we assessed if any of its downstream targets were affected in the mouse models. We therefore analyzed the expression level of pS6, a downstream target of mTORC1 but we did not detect any significant differences between the groups.

**Table 2:** Autophagy gene expression in rAAV-htt853 injected mice.

		rAAV5-htt853	
Gene	Uninjected controls	Q18	Q79
LC3B	1.00±0.08	1.00±0.08	0.78±0.05
LC3A	1.00±0.12	0.95±0.15	0.75±0.12
Atg5	1.00±0.11	1.19±0.17	0.90±0.13
Atg7	1.00±0.10	1.10±0.17	0.91±0.09
Beclin	1.00±0.10	1.14±0.16	0.85±0.14
p62	1.00±0.15	1.14±0.13	0.93±0.15
LAMP2	1.00±0.11	1.36±0.24	1.22±0.15
mTOR	1.00±0.14	0.80±0.12	0.59±0.09
Raptor	1.00±0.14	0.86±0.13	0.66±0.08
Rictor	1.00±0.10	1.11±0.19	0.92±0.15

Selected autophagy markers were analyzed by qRT-PCR in hypothalamic tissue from mice injected with rAAV5-htt853-79Q or rAAV5-htt853-18Q and uninjected controls, 8 weeks post injection (n = 7/group). The data in the table are expressed as mean fold change ± SEM, relative to the uninjected controls.

Furthermore, since p62 is known to be sequestered in mutant htt inclusions in animal models and human tissue, we used immunohistochemistry to analyze whether there would be p62 positive inclusions in rAAV-htt853 vector injected mice [65], [66], [67]. First we confirmed our previous findings of mutant htt inclusions in the hypothalamus of rAAV-htt853-79Q vector injected mice but not in the rAAV-htt853-18Q controls (Figure S1, A, B, A’, B’) [24]. Then we could identify p62 positive inclusions in the hypothalamus of rAAV-htt853-79Q but not in the uninjected side or in the rAAV-htt853-18Q controls (Figure S1, C, D). p62 aggregates were detected in some of the hypothalamic nuclei corresponding to the regions with highest density of mutant htt inclusions such as lateral hypothalamus and paraventricular nucleus (Figure S1, I, J) but not in other nuclei as arcuate nucleus and ventro-medial hypothalamus (Figure S1, K, L). No p62 positive inclusions were detected in the same regions in the rAAV-htt853-18Q controls (Figure S1, E–H).

Taken together, the data obtained suggest that although the overexpression of mutant htt can cause hypothalamic dysfunction with a strong metabolic phenotype, the molecular pathway of autophagy is preserved and the normal autophagic flux is unaltered in mice overexpressing htt fragments in the hypothalamus. To further confirm our data we decided to investigate the autophagy pathway in another HD mouse model.

### Analysis of the basal Levels of Autophagy in BACHD Hypothalami

**Figure 3:**
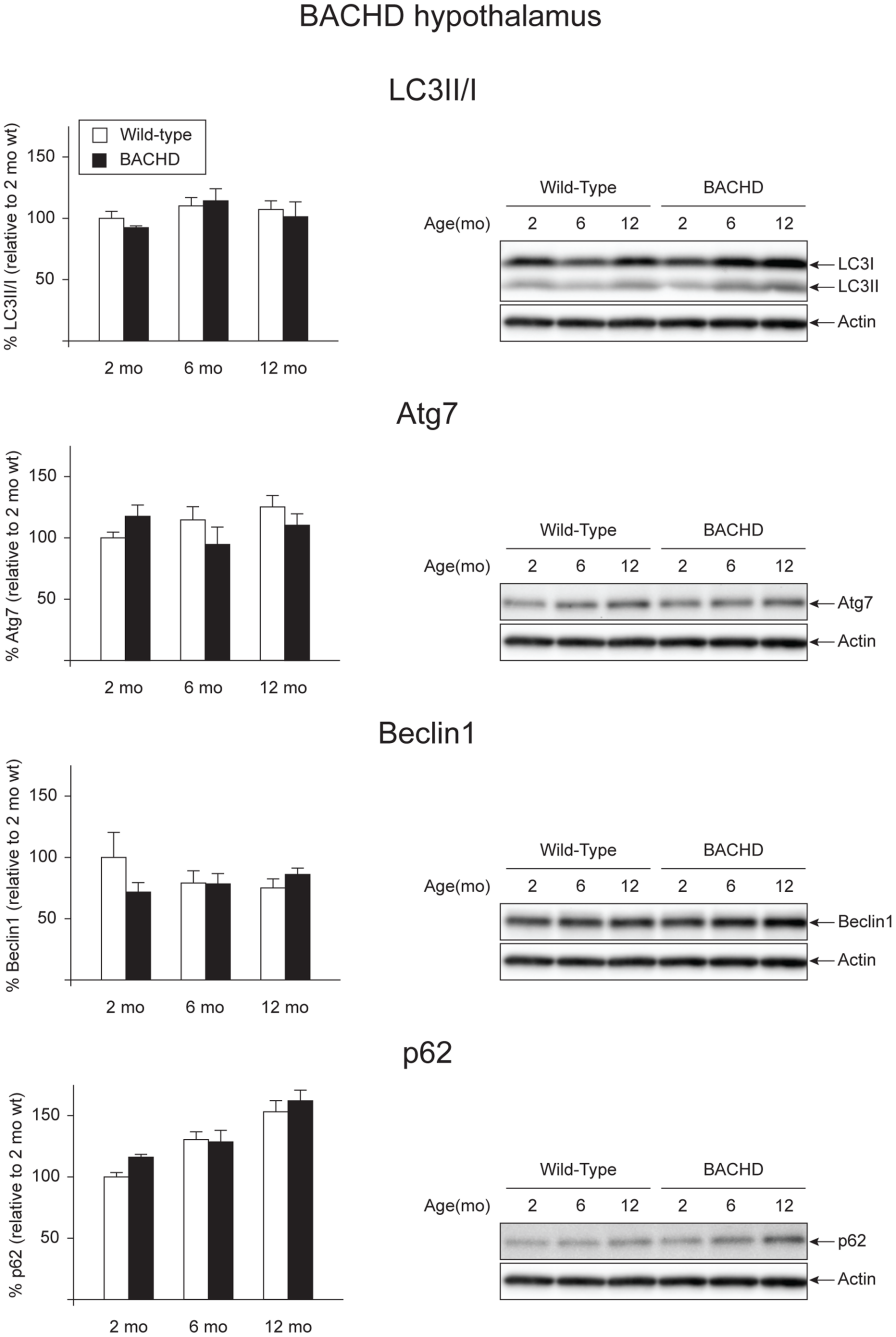
Basal levels of autophagy in the hypothalamus of BACHD mice. The expression levels of selected autophagy markers were analyzed by Western blot in hypothalamic tissue from BACHD mice at 2, 6 and 12 months of age and compared with gender- and age-matched controls (n = 3–6/group). The data showed a substantial maintenance of the autophagy flux, despite the presence of a metabolic phenotype in the BACHD mice already at early time points. The expression level of a marker for selective autophagy p62 showed a progressive accumulation in both BACHD and wt mice (Bonferroni post-hoc test: wt p = 0.003, BACHD p = 0.02). The data in the graphs are expressed as mean ± SEM, relative to the 2 months wt controls. The Western blots are representative of one sample for each group.

The results obtained from the analysis of the autophagy pathway in the rAAV5-htt853 vector injected mice suggested that expression levels of autophagy markers remained unaltered in the hypothalamus in the presence of overexpression of an 853 amino acid fragment of mutant htt. To further investigate whether basal levels of autophagy were affected in HD mouse models with metabolic dysfunction, we decided to perform further studies in a genetic, well-characterized model of HD, the BACHD mouse [26]. This HD mouse model expresses full-length human htt with 97Q and develops obesity as well as insulin and leptin resistance [24], [26]. The increase in body weight as well as in food intake can be observed already at 2 months of age and persists till 12 months of age in both female and male BACHD [24]. The expression of mutant htt in the cohort of samples used was validated by qRT-PCR and Western blot (Figure S2, A, B). We decided to investigate expression levels of markers of the autophagy pathway in hypothalamic tissue from female BACHD of 2, 6 and 12 months of age and in gender- and age-matched wt controls. qRT-PCR analysis of selected autophagy markers revealed changes in only some of the mRNA levels of the proteins considered. Expression levels of LC3B and LAMP2 showed genotype dependent significant differences which were not correlated with age (Genotype effect in two-factor ANOVA: LC3B: F_(1,21)_ = 6.451, p = 0.019; LAMP2: F_(1,21)_ = 5.704, p = 0.026) while Atg5 showed an increase in BACHD mice at 12 months of age (Genotype effect in two-factor ANOVA: F_(1,21)_ = 6.047, p = 0.023. Post-hoc un-paired Student T-test: p = 0.044) (Table 3). We observed an age-dependent decrease of expression levels of LC3A and p62 in qRT-PCR (Age effect in two-factor ANOVA: LC3A: F_(2,21)_ = 3.875, p = 0.037; p62: F_(2,21)_ = 6.848, p = 0.005; for more details see Statistical Results S1) (Table 3). Western blot analysis of selected autophagy markers didn’t reveal significant differences between BACHD and wt mice (n = 3–6/group) (Figure 3). However, we observed a progressive increase of p62 in both BACHD and wt mice, which is in accordance with previous studies (Age effect in two-factor ANOVA: F_(2,20)_ = 16.278, p<0.001; one-factor ANOVA followed by Bonferroni post-hoc test: wt p = 0.003, BACHD p = 0.02) [67]. The moderate effects observed in the BACHD hypothalamic region suggest that alterations in basal autophagy are not responsible for the hypothalamic dysfunction in this model.

**Table 3:** Autophagy gene expression in hypothalamic tissue of BACHD mice.

	WT			BACHD			
Gene	2 mo	6 mo	12 mo	2 mo	6 mo	12 mo	Stat
LC3B	1.00±0.10	0.91±0.05	0.90±0.05	1.10±0.04	1.06±0.06	1.00±0.03	*
LC3A	1.00±0.08	0.88±0.03	0.85±0.03	0.91±0.07	0.89±0.01	0.84±0.04	#
Atg5	1.00±0.05	0.98±0.02	0.85±0.09	1.04±0.10	1.16±0.09	1.08±0.04*	
Atg7	1.00±0.09	0.82±0.07	0.88±0.03	0.91±0.03	0.91±0.03	0.84±0.05	
Beclin	1.00±0.04	0.96±0.03	0.98±0.05	0.95±0.02	1.05±0.04	0.99±0.05	
p62	1.00±0.02	0.77±0.06	0.82±0.04	0.92±0.01	0.78±0.04	0.92±0.05	#
LAMP2	1.00±0.03	1.08±0.04	1.09±0.06	1.13±0.08	1.23±0.07	1.12±0.01	*
mTOR	1.00±0.10	0.94±0.05	0.91±0.04	0.87±0.00	0.91±0.04	0.91±0.02	
Raptor	1.00±0.03	0.99±0.03	0.93±0.06	0.93±0.03	0.88±0.05	0.90±0.02	
Rictor	1.00±0.07	1.06±0.05	0.96±0.06	0.99±0.05	0.89±0.16	1.10±0.02	

Selected autophagy markers analyzed by qRT-PCR in hypothalamic tissue from BACHD mice at 2, 6 and 12 months of age and compared with gender- and age-matched controls (n = 3–6/group). The data in the table are expressed as mean fold change ± SEM, relative to 2 months wt. Statistical significance was considered when p<0.05 and analysis were performed using two-factor ANOVA followed by post-hoc test or one-factor ANOVA followed by post-hoc test (*represents genotype effect while # represents age effect). For more detailed information see Statistical Results S1.

The increase in p62 levels might be related to the sequestration of p62 in mutant htt aggregates [67]. For this reason we tested whether we could detect both mutant htt and p62 inclusions in the hypothalamus of BACHD mice and wt controls at 12 months of age (Figure S3). However, using the same protocol as for the rAAV-htt853 vector injected animals, we did not detect any mutant htt inclusions nor p62 positive inclusions (Figure S3, A–D). The underlying mechanisms of hypothalamic dysfunction in HD remains elusive. It has previously been suggested that reduced expression of the transcription factor Brn-2 in another HD mouse model, the R6/2 mouse, could play a role [68]. Here we did not find any differences in Brn-2 mRNA expression levels in the hypothalamus of the two mouse models examined, which is in accordance with our previously published results using these models but assessed at other time points [24], [25] (Figure S4).

### Analysis of Basal Levels of Autophagy in the Cerebral Cortex and the Striatum of BACHD Mice

**Figure 4:**
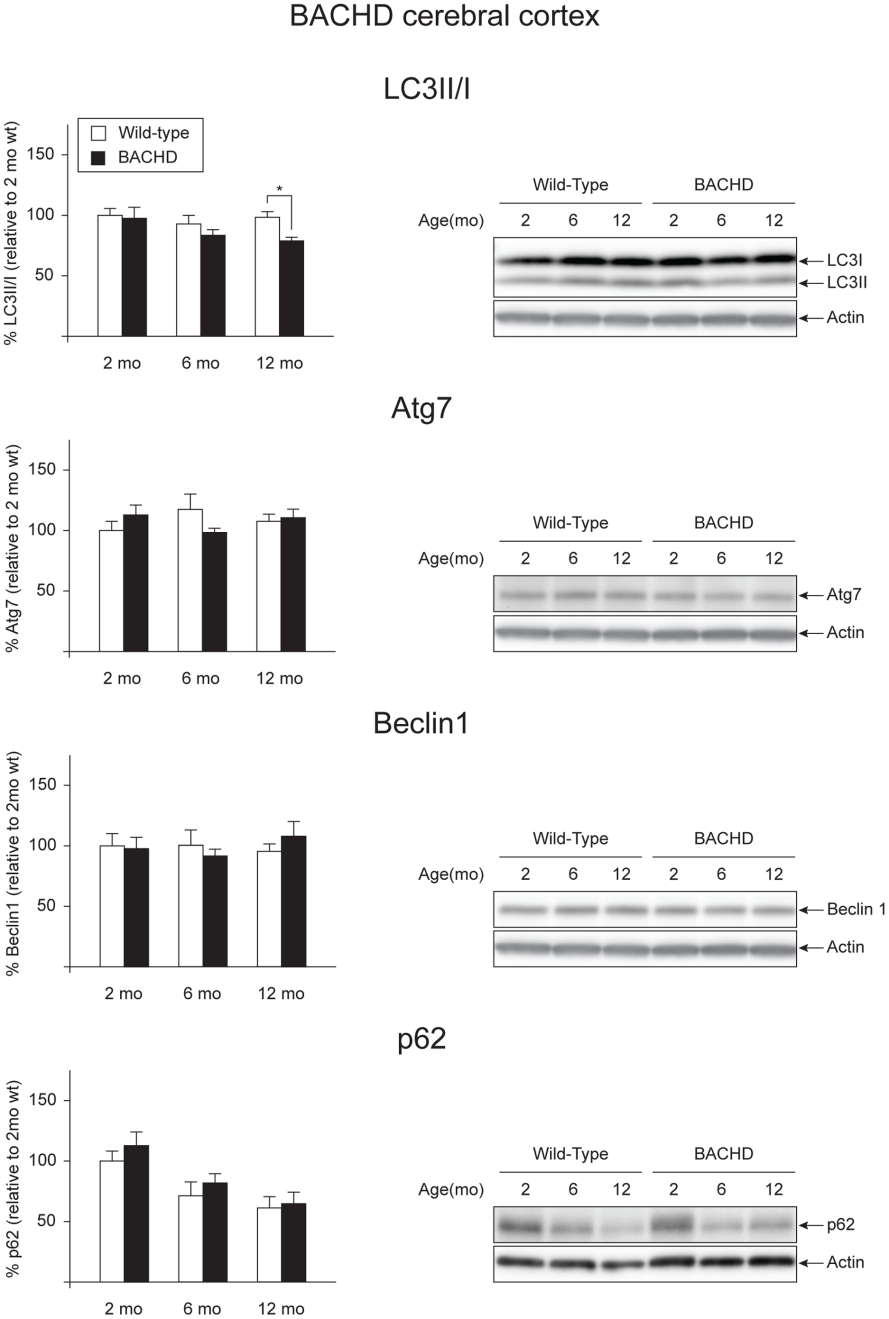
Basal levels of autophagy in the cerebral cortex of BACHD mice. The expression levels of selected autophagy markers were analyzed by Western blot in cortical tissue from BACHD mice at 2, 6 and 12 months of age and compared with gender- and age-matched controls (n = 3–6/group). The data show that the basal levels of autophagy were substantially unaltered in the cortex of BACHD mice with the exception of the LC3II/I ratio, which is reduced in 12 months old BACHD when compared with wt controls (Genotype effect in two-factor ANOVA: F_(1,25)_ = 4.715, p = 0.040; Student’s un-paired T-test as post hoc test: p = 0.006 at 12 months). The data in the graphs are expressed as mean ± SEM, relative to the 2 months wt controls. The Western blots are representative of one sample for each group.

**Figure 5:**
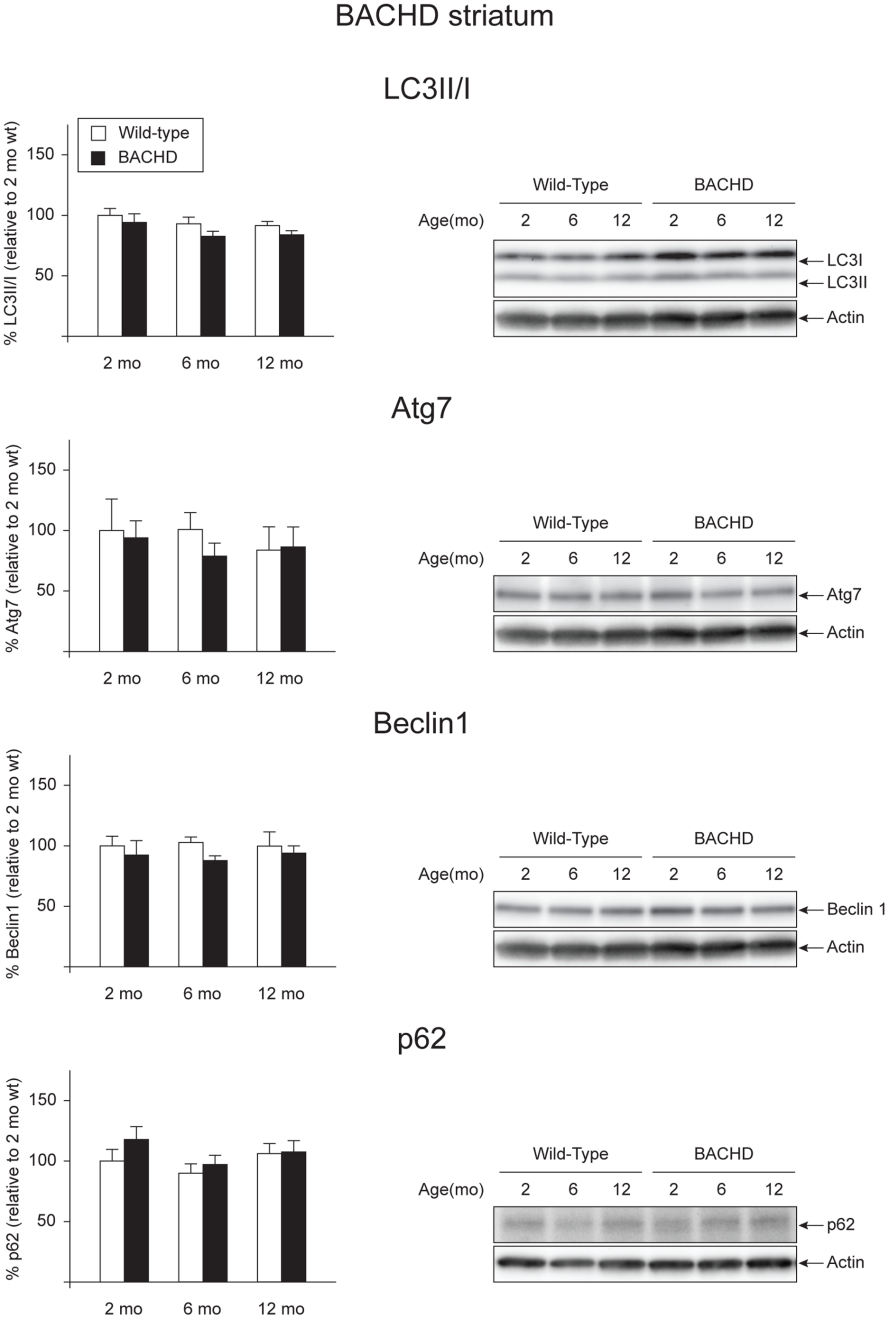
Basal levels of autophagy in the striatum of BACHD mice. Selected autophagy markers were analyzed by Western blot of striatal tissue from BACHD mice at 2, 6 and 12 months of age and compared with gender- and age-matched controls (n = 3–6/group). The data show that the basal levels of autophagy were unaltered in the striatum of BACHD mice. The data in the graphs are expressed as mean ± SEM, relative to the 2 months wt controls. The Western blots are representative of one sample for each group.

BACHD mice ubiquitously express human full-length mutant htt, thus allowing us to study other brain regions important for HD pathology such as cerebral cortex and striatum [26], [69], [70], [71]. Although the main aim of the project was to investigate whether alterations in autophagy could be a potential contributor to hypothalamic dysfunction in HD, we were also interested in characterizing the autophagic pathway in two other brain regions known to play an important role in disease progression [4], [69], [70], [72], [73]. Furthermore, it is thought that regulation of feeding behavior might in part be dependent on corticostriatal-hypothalamic projections, thus changes in these two regions might influence the hypothalamus and its functional circuitries, compromising normal feeding behavior [74], [75]. We therefore investigated expression levels of several factors in the autophagy pathway at basal conditions in these two brain areas in BACHD mice. The expression of mutant htt in the cohort of samples was validated by qRT-PCR and Western blot (Figure S2 C–F). We performed qRT-PCR on one hemisphere of cerebral cortex and striatum of BACHD animals at 2, 6 and 12 months of age and gender- and age-matched wt mice for selected autophagy markers. We found a significant reduction of Atg7 in the cerebral cortex of BACHD mice at 12 months of age when compared with the wt controls (Genotype effect in two-factor ANOVA: F_(1,25)_ = 14.233, p = 0.001, Student’s un-paired T-test as post hoc test p = 0.006) (Table 4). Furthermore, we found that Beclin1 appeared to follow an age dependent decrease (Age effect in two-factor ANOVA: F_(2,25)_ = 3.900, p = 0.034) (Table 4). In the striatum, the only autophagy component changed among those analyzed was p62, which was increased both at 6 and 12 months, when compared to wt controls (Genotype effect in two-factor ANOVA: F_(1,25)_ = 17.301, p<0.001; Student’s un-paired T-test as post hoc test: p = 0.021 and p = 0.009 at 6 and 12 months of age respectively) (Table 5). Consistently with our results in the hypothalamus, the quantification of the bands in the Western blot revealed a general maintenance of the basal autophagy levels both in the cerebral cortex and the striatum (Figure 4 and 5). Notably, at 12 months of age, the LC3II/I ratio in cortical tissue from BACHD mice was reduced compared to wt controls, which could indicate a reduced activation of autophagy flux in this region (Genotype effect in two-factor ANOVA: F_(1,25)_ = 4.715, p = 0.040; Student’s un-paired T-test as post hoc test: p = 0.006 at 12 months) (Figure 4) [76]. On the contrary to what was found in the hypothalamic tissue, the levels of p62 decreased over time in the cerebral cortex of BACHD mice as well as in wt animals (age effect in two-factor ANOVA: F_(2,24)_ = 8.329, p = 0.002; Bonferroni post-hoc test p = 0.013 between 2 and 12 months of age). Similar changes weren’t observed in the striatum.

**Table 4:** Autophagy gene expression in cortical tissue of BACHD mice.

	WT			BACHD			Stat
Gene	2 mo	6 mo	12 mo	2 mo	6 mo	12 mo	
LC3B	1.00±0.07	0.99±0.03	0.94±0.03	0.91±0.05	0.93±0.02	0.90±0.02	
LC3A	1.00±0.04	1.02±0.04	1.01±0.03	1.10±0.03	1.09±0.07	0.97±0.03	
Atg5	1.00±0.05	1.04±0.05	1.03±0.02	1.06±0.04	1.00±0.05	1.01±0.05	
Atg7	1.00±0.02	0.99±0.03	1.06±0.03	0.93±0.04	0.92±0.03	0.88±0.04*	
Beclin	1.00±0.04	0.98±0.03	0.87±0.01	1.02±0.03	0.98±0.03	0.95±0.05	#
p62	1.00±0.09	1.05±0.05	0.98±0.05	1.16±0.03	1.02±0.03	1.10±0.08	
LAMP2	1.00±0.03	1.13±0.07	1.07±0.02	1.03±0.04	1.01±0.03	1.02±0.03	
mTOR	1.00±0.11	1.11±0.03	1.21±0.07	1.10±0.05	1.15±0.11	1.00±0.07	
Raptor	1.00±0.07	1.10±0.04	1.09±0.05	1.04±0.04	1.10±0.07	0.95±0.03	
Rictor	1.00±0.04	1.09±0.02	1.02±0.05	0.97±0.03	1.00±0.06	1.03±0.05	

Selected autophagy markers analyzed by qRT-PCR in tissue from cerebral cortex from BACHD mice at 2, 6 and 12 months of age and compared with gender- and age-matched controls (n = 3–6/group). The data in the table are expressed as mean fold change ± SEM, relative to 2 months wt. Statistical significance was considered when p<0.05 and analysis were performed using two-factor ANOVA followed by post-hoc test or one-factor ANOVA followed by post-hoc test (*represents genotype effect while # represents age effect). For more detailed information see Statistical Results S1.

**Table 5:** Autophagy gene expression in striatal tissue of BACHD mice.

	WT			BACHD		
Gene	2 mo	6 mo	12 mo	2 mo	6 mo	12 mo
LC3B	1.00±0.04	0.88±0.07	0.91±0.05	0.92±0.06	0.91±0.03	0.89±0.05
LC3A	1.00±0.03	1.02±0.09	1.00±0.02	1.13±0.03	1.04±0.06	1.11±0.04
Atg5	1.00±0.04	0.99±0.06	0.98±0.05	1.07±0.01	1.08±0.05	1.07±0.06
Atg7	1.00±0.04	1.15±0.09	1.11±0.08	1.02±0.02	1.15±0.08	1.14±0.06
Beclin	1.00±0.05	0.94±0.05	0.94±0.05	1.03±0.03	0.98±0.05	0.93±0.03
p62	1.00±0.03	0.93±0.03	0.90±0.05	1.06±0.04	1.05±0.02*	1.12±0.05*
LAMP2	1.00±0.09	0.85±0.07	0.89±0.10	0.81±0.10	0.83±0.07	0.86±0.06
mTOR	1.00±0.08	0.97±0.06	0.99±0.10	0.84±0.05	1.02±0.05	1.08±0.13
Raptor	1.00±0.04	0.93±0.04	1.00±0.12	0.94±0.07	0.95±0.07	0.95±0.08
Rictor	1.00±0.04	1.12±0.11	1.23±0.14	1.13±0.09	1.23±0.14	1.12±0.08

Selected autophagy markers analyzed by qRT-PCR in tissue from striata from BACHD mice at 2, 6 and 12 months of age and compared with gender- and age-matched controls (n = 3–6/group). The data in the table are expressed as mean fold change ± SEM, relative to 2 months wt. Statistical significance was considered when p<0.05 and analysis were performed using two-factor ANOVA followed by post-hoc test or one-factor ANOVA followed by post-hoc test (*represents genotype effect). For more detailed information see Statistical Results S1.

## Discussion

Individuals with HD often develop non-motor symptoms and signs several years before the onset of motor disturbances. Changes in metabolism in experimental models of HD have recently gained significant attention [24], [52], [53], [77], [78]. Despite a sometimes severe weight loss at late stages of the disease, HD patients have been described to display increased appetite [79], [80], insulin resistance, alteration in leptin levels and higher susceptibility to develop diabetes [10], [12], [81], [82]. Metabolic dysfunction with obesity and insulin as well as leptin resistance can be observed in genetic models of HD expressing human full-length htt, such as the YAC and BACHD mice [24], [26], [83]. A similar metabolic phenotype could also be induced by selective overexpression of mutant htt fragments in the hypothalamus of wt mice, and inactivation of mutant htt in the hypothalamus of BACHD mice prevented the development of metabolic disturbances [24]. These data suggest that hypothalamic dysfunction may be involved in the metabolic dysfunction in HD. The molecular mechanisms by which mutant htt disrupts hypothalamic circuitry involved in metabolic control is not yet known.

Autophagy is a degradation pathway responsible of the clearance of misfolded proteins, organelles and aggregates [28]. Recent studies have showed that this pathway is important for the regulation of food intake and metabolism in the hypothalamus [44], [45], [46], [47], [84]. Inhibition of autophagy in a hypothalamic neuronal population expressing agouti-related peptide (AgRP) neurons through the knock down of one of the essential components in autophagy, Atg7, reduces food intake and adiposity [45]. On the contrary, knock down of Atg7 in the mediobasal hypothalamus as well as in propriomelanocortin (POMC) neurons, favors the onset of an obesity phenotype and leptin resistance [44], [46], [47]. Also, p62 has been found to be enriched in the hypothalamus in mice, and brain-specific knock out of p62 leads to hyperphagia and leptin resistance [48]. Interestingly, there is evidence suggesting that autophagy plays a crucial role in neurodegenerative disorders, since silencing of essential components of the autophagy pathway induce neuronal degeneration in mouse models [32], [85], [86], [87]. Also, autophagy is thought to have an important role in polyglutamine dirorders and paticularly in the pathogenesis of HD [28], [33], [88]. Autophagy is in fact thought to contribute to the degradation of mutant and wt htt as well as to the processing of mutant htt fragments [34], [35], [36], [37]. Expression of htt has also been shown to induce the activation of the lysosomal system and autophagy in striatal cell lines [35]. Similarly, the presence of mutant htt fragments activates dopamine-mediated autophagy in cells derived from the R6/2 HD model [31]. Furthermore, activation of autophagy and improvement of HD phenotypes have been shown in an HD mouse model expressing mutant htt with a deletion of the polyglutamine stretch [38]. Finally, manipulation of the autophagic pathway has been shown to improve the neuropathology and the behavioral phenotypes in experimental models of HD [39], [41], [89], [90].

Based on the role of autophagy in hypothalamic-mediated obesity as well as its potential involvement in HD pathogenesis, we investigated whether the autophagic pathway was affected in the hypothalamus of two mouse models of HD with metabolic dysfunction. We measured basal levels of a number of candidate autophagic markers using both Western Blot and qRT-PCR in the hypothalami from these mice. For the mice with selective hypothalamic expression of htt fragments, we performed the analyses at 8 weeks post-injection when the metabolic changes were present but still developing. For the BACHD mice, we analyzed hypothalamic tissue both at 2 months of age when metabolic features were present, as well as at two older ages when the metabolic disturbances were fully manifest and constant. We decided not to challenge the pathway by using compounds inducing autophagy, such as rapamycin or other mTOR inhibitors, although these were previously used to demonstrate positive effects on htt clearance and behavioral improvements in HD models [39], [41], [91], [92]. Our aim was instead to evaluate whether presence of mutant htt in the hypothalami of BACHD or rAAV5-htt853 vector injected animals would be sufficient to modify the flux of autophagy in the hypothalamic neurocircuitry. The data obtained suggest that expression of mutant htt is not sufficient to induce dramatic changes in basal autophagy in the hypothalamus, indicating that this pathway is unlikely to be involved in hypothalamic dysfunction and consequent metabolic signs in HD.

In this study, we assessed a selected number of well-characterized markers in the complex autophagy pathway. At the protein level, we first decided to evaluate the conversion of LC3I in LC3II. This event is often used to indicate changes in the autophagic flux, although monitoring these alterations *in vivo*, especially in neuronal cells, might be more difficult than in *in vitro* cellular models [76], [93]. The hypothalamic tissue analyzed in this study showed that no significant changes occurred in the autophagy flux since LC3II/I ratio remained constant. These data suggest instead maintenance of the basal level of autophagy as well as a normal level of autophagosome formation both in rAAV injected mice 8 weeks post injection and in BACHD mice at 2 months of age. This effect remained stable also when we studied BACHD mice at later time points (6 and 12 months of age). Interestingly, we found a significant reduction in LC3II/I ratio in the cerebral cortex of BACHD mice at 12 months of age, indicating that cortical neurons were not able to successfully counteract the presence of mutant htt at that stage. Other studies showed both an early induction of autophagy in HdhQ200 mice as well as alterations in cargo recognition in HdhQ111 mice [33], [94]. Although our results are contrasting with these data, one has to consider that none of these studies specifically investigated hypothalamic pathology and our experimental techniques as well as our animal models differ from those previously used.

We also analyzed expression levels of Atg7 and Beclin1 using Western Blot. Atg7 is an essential component of the autophagy pathway, contributing to LC3I lipidation into LC3II [95]. Deficiency in this marker compromise the pathway and have been thought to induce neurodegeneration in mice as well as inducing metabolic disturbances when knocked down in the hypothalamus [44], [45], [46], [47], [85], [96]. Furthermore a specific polymorphism of this gene may be a modifier of age of onset in HD [97]. On the other hand the expression levels of Beclin1, another crucial component of the autophagy pathway, may regulate the accumulation of mutant htt fragments [98], [99]. When analyzing both these markers in the hypothalamic region of BACHD and rAAV5-htt853 vector injected mice, we didn’t detect any differences between the genotypes, suggesting that this region might require stronger stimuli then just the presence of mutant htt to compromise the autophagy pathway. Surprisingly we did not detect any significant differences in these markers in the cerebral cortex and striatum of BACHD mice.

p62 is another important component of the selective autophagy pathway and it is normally responsible of targeting ubiquitinated proteins to the lysosomes as well as being a substrate of autophagy itself [62]. Furthermore p62 has been shown to co-localize with mutant htt aggregates both *in vivo* and *in vitro* and to accumulate in age dependent fashion [65], [66], [67]. Although in our study we did not detect any difference in p62 levels among control animals, rAAV-htt853-18Q and rAAV-htt853-79Q, we did observe an age dependent increase of p62 levels in the hypothalamus of both BACHD and wt animals. These results are at least partially in accordance with previous published results, although in other studies the age dependent accumulation was found only in the R6/1 HD model and not in the wt controls [67]. Surprisingly, the levels of p62 were progressively reduced in cerebral cortex but not striatum of BACHD mice and controls, which might indicate that changes are occurring in the degradation rate of the protein.

We have also analyzed relevant autophagy markers and components of related pathways using qRT-PCR. Transcriptional dysregulation is a hallmark of HD and significant changes in gene transcription of key intracellular pathways have been found in both clinical HD material and several HD mouse models [51], [52], [53], [54], [55], [56], [57], [58]. Besides LC3, Atg7, Beclin1 and p62, we investigated Atg5, another key component of the autophagosome formation, and LAMP2, an important lysosomal membrane bound protein, involved mainly in chaperone mediate autophagy (CMA) [100]. Interestingly, an upregulation of CMA has been shown in the ^111^QHtt HD model, with increased mRNA levels of LAMP2 [101]. We also investigate mRNA levels of several components of the mTOR pathway, which acts as an upstream negative regulator of autophagy. mTOR is thought to inhibit mainly starvation induced autophagy and compounds acting on this pathway have been shown to contribute to the clearance of htt aggregates and to improve symptoms in several HD models [39], [40]. Although there were no significant differences between the groups of any of the markers, we found a trend for reduction for mTOR (p = 0.085) in the AAV5-htt853-79Q group compared to the AAV5-htt853-18Q group and controls. In order to verify if this trend could result in a differential activation of the pathway, we decided to explore downstream components of the mTORC1, which are activated by post-translational modifications. To this aim we chose pS6, known to be substrate of S6 kinase, direct target of mTORC1 [102]. However the high variability of the phosphorylated levels of S6 didn’t allow us to reach conclusive results. Interestingly, when we analyzed the same markers in the BACHD hypothalamus we found that genotype had an effect on LC3B, Atg5 and LAMP2, which resulted in increased expression levels in the BACHD mice. The downregulation of p62 is not consistent with the progressive accumulation seen at the protein levels, however compensatory mechanisms might occur which prevent further transcription of the mRNA in presence of already elevated levels of the protein. In the cerebral cortex we found that the levels of Atg7 were reduced at 12 months in BACHD compared to wt and that Beclin was progressively reduced over time in both genotypes. Finally in the striatum we could only observe a genotype dependent increase in p62 mRNA at 6 and 12 months of age.

The underlying molecular and cellular mechanisms of hypothalamic dysfunction and metabolic disturbances in HD are not yet known. A number of factors have been suggested to play a role but the results are sometimes conflicting [19]. Reduced expression and activity of the transcription factor Brn-2 has been found in the hypothalamus of the R6/2 mouse, but not in the mouse models used here [24], [25], [68]. Although the metabolic regulator orexin is reduced in the hypothalamus of clinical HD and in the rAAV model, the number of the orexin immunopositive cells are in fact increased but atrophied in BACHD mice [21], [22], [23], [24], [25]. Furthermore, inactivation of mutant htt in leptin receptor expressing neurons in this mouse had no effect on the metabolic phenotype [103]. However, the BACHD with the possibility to delete mutant huntingtin in specific neuronal populations using cre-recombinase technology remains an interesting model to further explore the underlying substrate for this phenotype [26].

In conclusion, the data reported in this study suggest that expression of mutant htt alone is not sufficient to alter the basal levels of autophagy in the studied HD mouse models. Alterations in the autophagic pathway are therefore unlikely to be involved in the hypothalamic dysfunction and the development of metabolic disturbances in these HD mouse models. Furthermore, only mild changes were found in the autophagic pathway in the cerebral cortex and the striatum of BACHD mice at older age, indicating that autophagy is rather well maintained through out the disease progression in the mice. It remains possible that the mice would differentially respond to a challenge of the autophagic pathway such as starvation or a compound dependent inhibition of mTOR. It is also possible that presence of mutant htt might differentially affect the several neuronal populations constituting the complex hypothalamic neurocircuitry for control of food intake and metabolism. Further studies would be needed to address these interesting questions.

## Supporting Information

Figure S1 **Histological analysis of htt inclusions and p62 in the hypothalamus of mice unilaterally injected with rAAV5-htt853 vectors.** Mutant htt inclusions were detected in the hypothalamus of mice unilaterally injected with rAAV5-htt853-79Q (B, B’) but not in the hypothalamus of AAV5-htt853-18Q (A, A’) mice. p62 positive inclusions were also detected in the hypothalamus of mice injected with rAAV5-htt853-79Q (D) but only diffused staining could be detected when rAAV5-htt853-18Q was injected (C). In particular, in rAAV5-htt853-79Q vector injected mice, p62 positive inclusions were visible in the lateral hypothalamus (LH) and in the paraventricular nucleus (PVN), but to less a extent in the arcuate nucleus (ARC) and in the ventromedial hypothalamus (VMH) (I-L). Only diffuse staining could be detected in the corresponding areas in AAV5-htt853-18Q vector injected animals. Scale bar in (D), 200 µm and applies to (A,B,C); in (L), 25 µm and applies to (A’, B’ and E–K).
(TIF)

Figure S2 **Expression levels of wt and mutant htt in BACHD mice.** qRT-PCR (A, C, E) and Western blot (B, D, F) analysis of the expression levels of wt and mutant htt in the hypothalamus, cerebral cortex and striatum of BACHD mice. The qRT-PCR data are expressed as mean ± SEM and were calculated as relative to the 2 mo wt. The Western blots are representative of one sample per group and they were performed using the MAB2166 antibody which recognizes both forms of the htt protein.
(TIF)

Figure S3 **Histological analysis of htt inclusions and p62 in the hypothalamus of BACHD mice.** Htt expression in the hypothalamus of BACHD mice and wt controls at 12 months of age (A, B). The hypothalamic region of BACHD mice did not display detectable mutant htt inclusions (B). p62 positive cells could be detected in the hypothalamus of wt and BACHD mice without presence of p62 inclusions (C, D). Scale bar in (D), 200 µm and applies to (A,B,C).
(TIF)

Figure S4 **Similar expression levels of Brn-2 in the hypothalamus of AAV5-htt853 vector injected and BACHD mice compared to their controls.** qRT-PCR analysis of the expression levels of the transcription factor Brn-2 in the hypothalamus of rAAV5-htt853 vector injected (A) and BACHD (B) mice. The data are expressed as mean ± SEM and were calculated as relative to control (A) and 2 mo wt (B). The primers used for the qRT-PCR were the following (5′-3′): forward primer ATGGCGACCGCAGCGTCTAAC, reverse primer AGGCGGCTCGGCATGTACGA.
(TIF)

Table S1 **Primer sequences used in qRT-PCR.**
(DOCX)

Material and Methods S1 (DOCX)

Statistical Results S1 (DOCX)
