# Supplementary figures and images for: Maintenance of Basal Levels of Autophagy in Huntington’s Disease Mouse Models Displaying Metabolic Dysfunction
Source: PLoS One. 2013 Dec 20;8(12):e83050. doi: 10.1371/journal.pone.0083050 (PMC3869748; doi:10.1371/journal.pone.0083050)

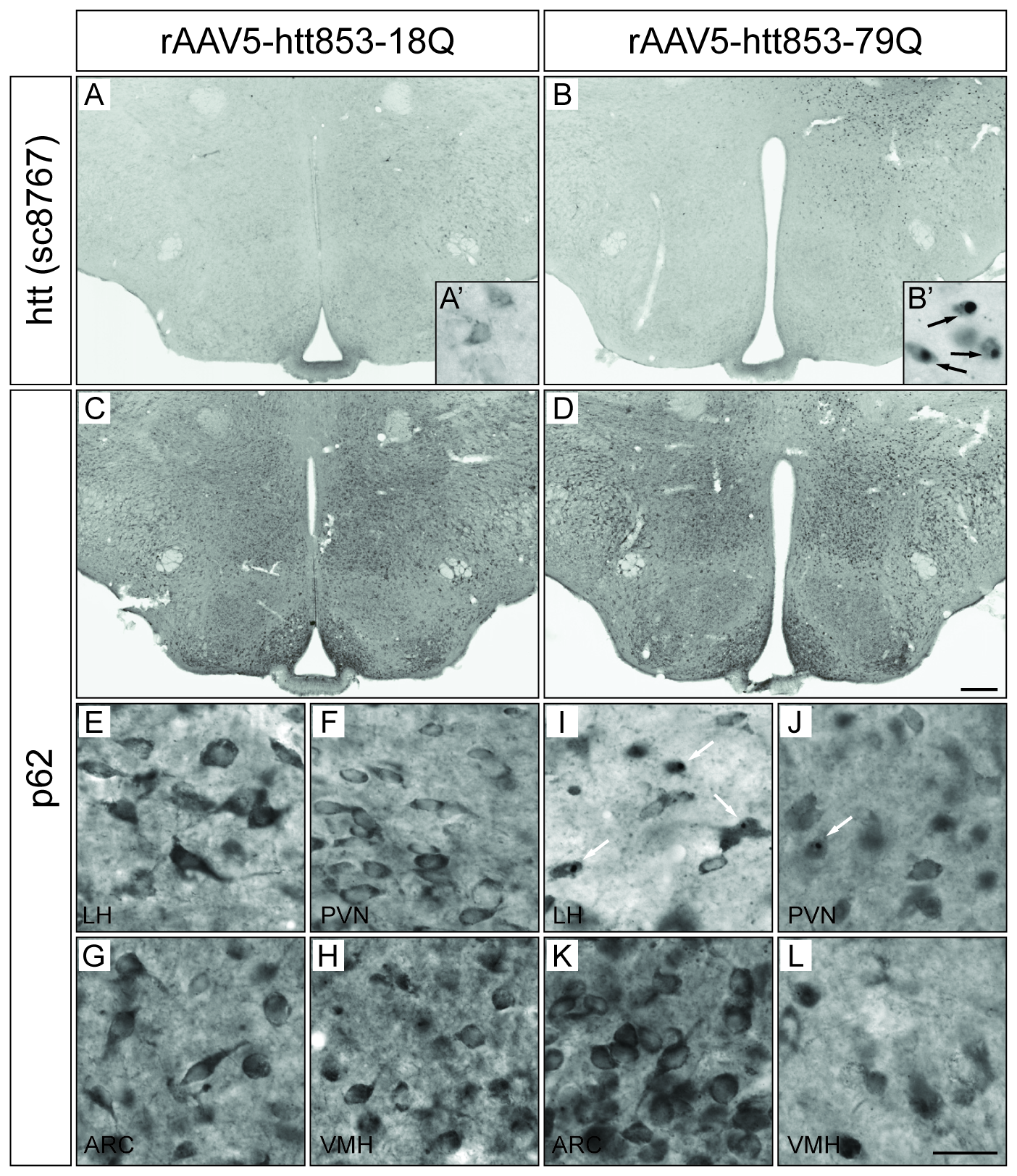

Supplement: Figure S1 — Histological analysis of htt inclusions and p62 in the hypothalamus of mice unilaterally injected with rAAV5-htt853 vectors. Mutant htt inclusions were detected in the hypothalamus of mice unilaterally injected with rAAV5-htt853-79Q (B, B’) but not in the hypothalamus of AAV5-htt853-18Q (A, A’) mice. p62 positive inclusions were also detected in the hypothalamus of mice injected with rAAV5-htt853-79Q (D) but only diffused staining could be detected when rAAV5-htt853-18Q was injected (C). In particular, in rAAV5-htt853-79Q vector injected mice, p62 positive inclusions were visible in the lateral hypothalamus (LH) and in the paraventricular nucleus (PVN), but to less a extent in the arcuate nucleus (ARC) and in the ventromedial hypothalamus (VMH) (I-L). Only diffuse staining could be detected in the corresponding areas in AAV5-htt853-18Q vector injected animals. Scale bar in (D), 200 µm and applies to (A,B,C); in (L), 25 µm and applies to (A’, B’ and E–K). (TIF) [file pone.0083050.s001.tif]

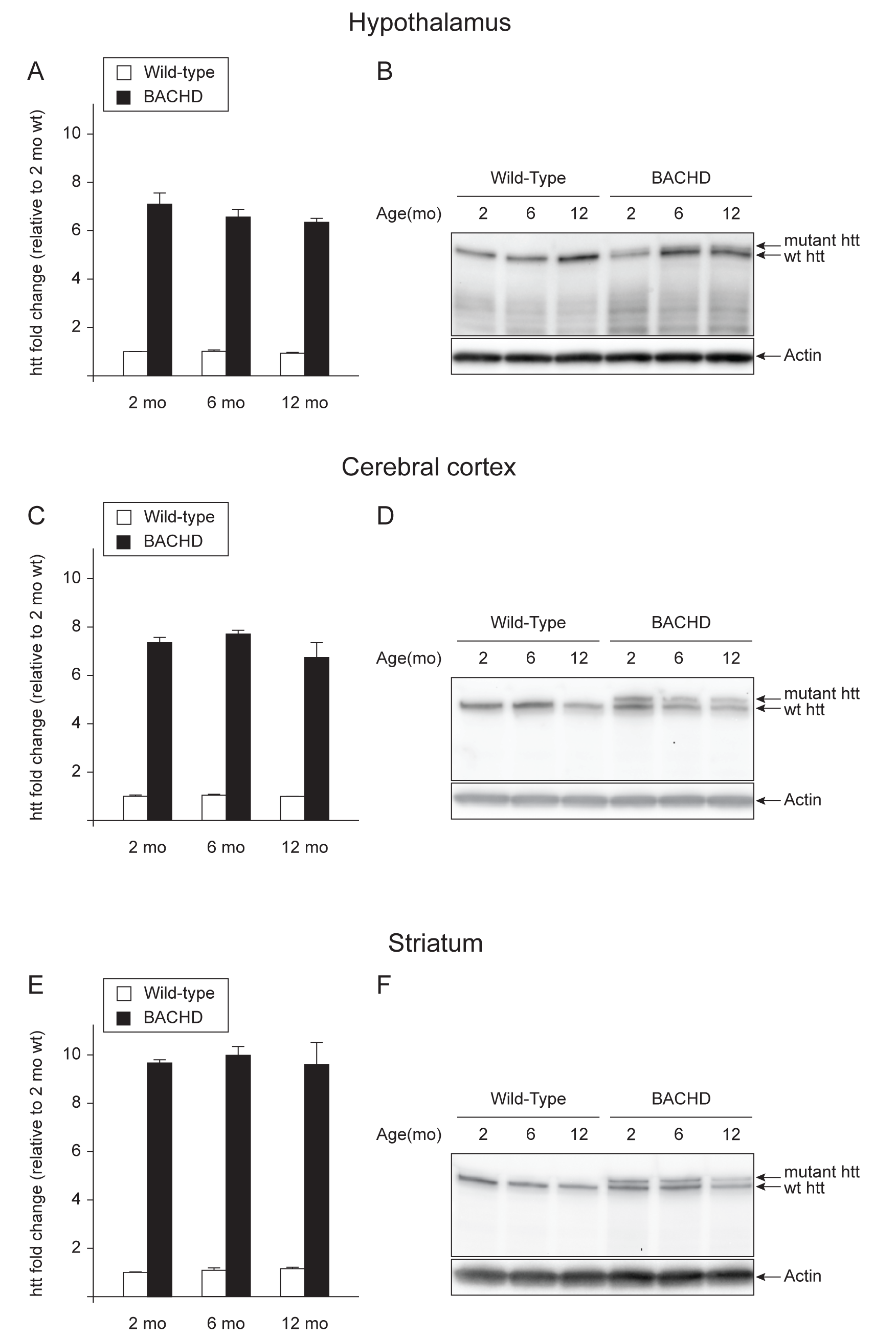

Supplement: Figure S2 — Expression levels of wt and mutant htt in BACHD mice. qRT-PCR (A, C, E) and Western blot (B, D, F) analysis of the expression levels of wt and mutant htt in the hypothalamus, cerebral cortex and striatum of BACHD mice. The qRT-PCR data are expressed as mean ± SEM and were calculated as relative to the 2 mo wt. The Western blots are representative of one sample per group and they were performed using the MAB2166 antibody which recognizes both forms of the htt protein. (TIF) [file pone.0083050.s002.tif]

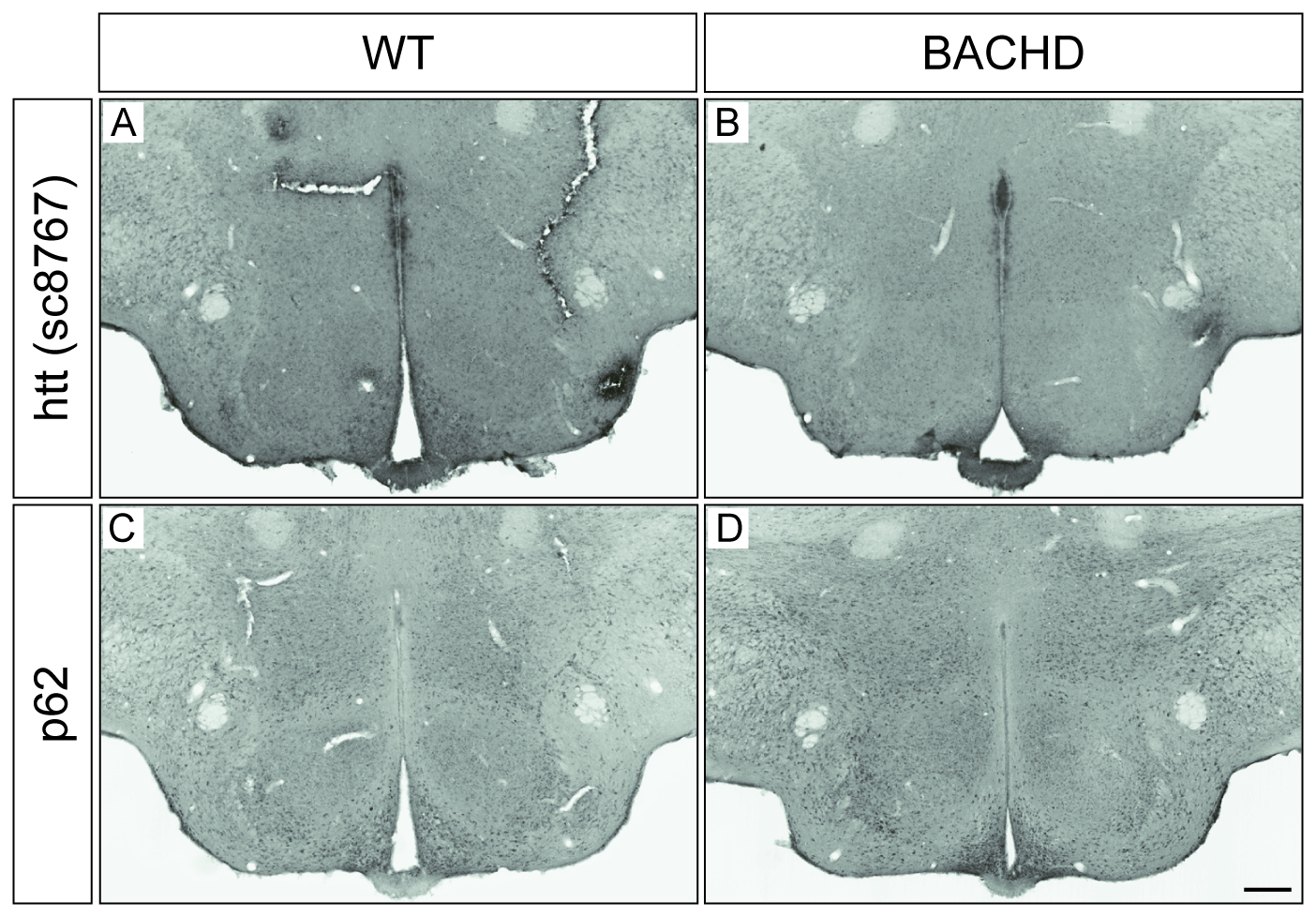

Supplement: Figure S3 — Histological analysis of htt inclusions and p62 in the hypothalamus of BACHD mice. Htt expression in the hypothalamus of BACHD mice and wt controls at 12 months of age (A, B). The hypothalamic region of BACHD mice did not display detectable mutant htt inclusions (B). p62 positive cells could be detected in the hypothalamus of wt and BACHD mice without presence of p62 inclusions (C, D). Scale bar in (D), 200 µm and applies to (A,B,C). (TIF) [file pone.0083050.s003.tif]

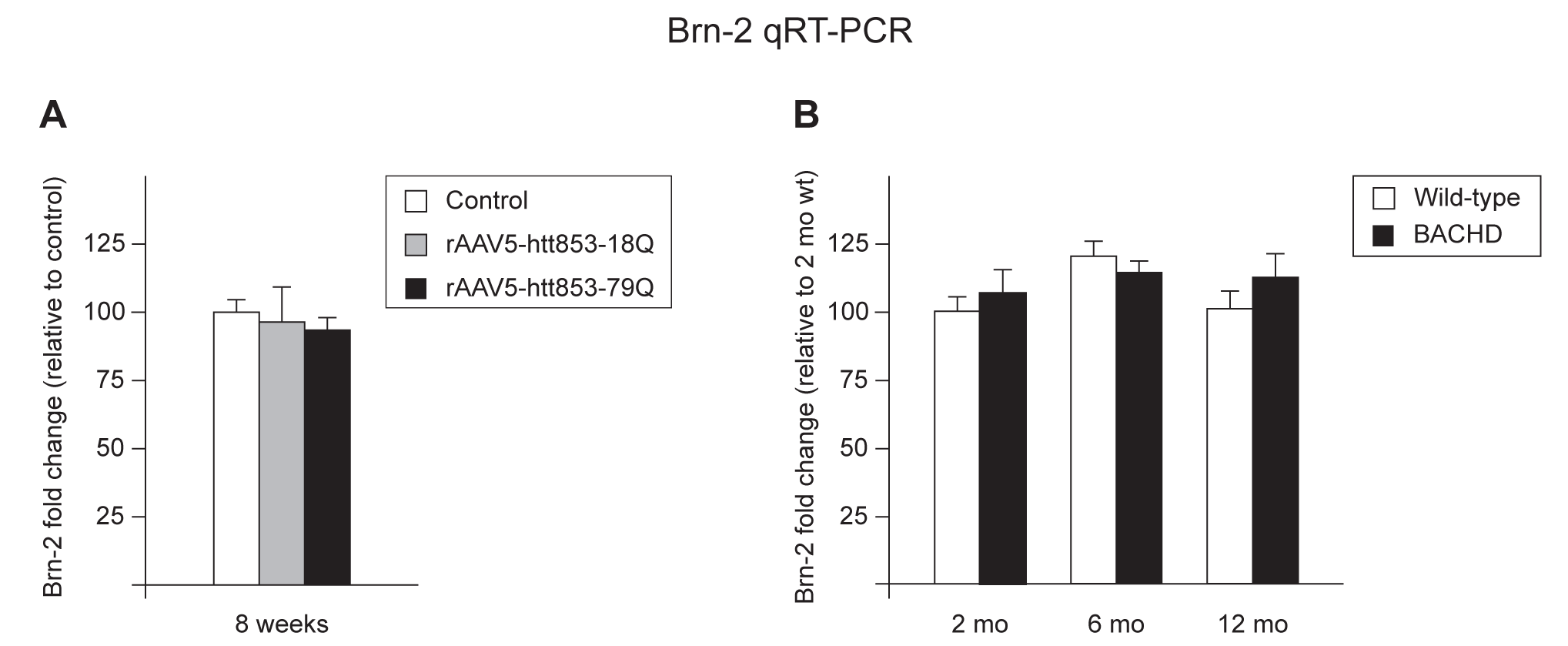

Supplement: Figure S4 — Similar expression levels of Brn-2 in the hypothalamus of AAV5-htt853 vector injected and BACHD mice compared to their controls. qRT-PCR analysis of the expression levels of the transcription factor Brn-2 in the hypothalamus of rAAV5-htt853 vector injected (A) and BACHD (B) mice. The data are expressed as mean ± SEM and were calculated as relative to control (A) and 2 mo wt (B). The primers used for the qRT-PCR were the following (5′-3′): forward primer ATGGCGACCGCAGCGTCTAAC, reverse primer AGGCGGCTCGGCATGTACGA. (TIF) [file pone.0083050.s004.tif]
