# Supplementary material for: Maintenance of Basal Levels of Autophagy in Huntington’s Disease Mouse Models Displaying Metabolic Dysfunction
Source: PLoS One. 2013 Dec 20;8(12):e83050. doi: 10.1371/journal.pone.0083050 (PMC3869748; doi:10.1371/journal.pone.0083050)
Supplement: Table S1 — Primer sequences used in qRT-PCR. (DOCX) [file pone.0083050.s005.docx]

**Table S1. Primer sequences used in qRT-PCR.**

|  | **Primers 5'- 3'** | |
| --- | --- | --- |
| **Gene** |  |  |
| LC3B | Forward | TGTGTAACTGTCTCTGTAAG |
|  | Reverse | TCTTCTGTTGCTGTTGTC |
| LC3A | Forward | CTATGAACAGGAGAAGGATGAAG |
|  | Reverse | ACTCAGAAGCCGAAGGTT |
| Atg5 | Forward | GGTTATGAGACAAGAAGATG |
|  | Reverse | TACTGTGATGTTCCAAGG |
| Atg7 | Forward | CCAGAAGAAGTTGAACGAGTA |
|  | Reverse | CCAGCAGAGTCACCATTG |
| Beclin | Forward | AGCGGGAGTATAGTGAGTTTA |
|  | Reverse | AAGGTGGCATTGAAGACATT |
| p62 | Forward | CCTACAGACCAAGAATTACGA |
|  | Reverse | GCCACAGCACTATCACAA |
| LAMP2 | Forward | CCACTCCAACTCCAACTC |
|  | Reverse | CCAGCAGACAGGTAGTATTG |
| mTor | Forward | AGCAACAGTGAGAGTGAAG |
|  | Reverse | CAAGGAGATAGAACGGAAGAA |
| Raptor | Forward | GGACCTTACAGATTGGAACT |
|  | Reverse | CACACTCACCGTCTTCAT |
| Rictor | Forward | GAGGTGATAACGAACAGTGA |
|  | Reverse | TGGCAAGCAGTGTAGATG |
