# Supplementary material for: Maintenance of Basal Levels of Autophagy in Huntington’s Disease Mouse Models Displaying Metabolic Dysfunction
Source: PLoS One. 2013 Dec 20;8(12):e83050. doi: 10.1371/journal.pone.0083050 (PMC3869748; doi:10.1371/journal.pone.0083050)
Supplement: Material and Methods S1 — (DOCX) [file pone.0083050.s006.docx]

**Material and methods S1**

**Immunohistochemistry**

BACHD mice were anesthetized at 12 months of age while mice injected unilaterally with rAAV5-htt853 were anesthetized 6 weeks post injection with sodium pentobarbital (Apoteksbolaget). The animals were then transcardially perfused with ice-cold 4% paraformaldehyde (PFA). The brains were collected and post-fixed in PFA for 24 hours before cryoprotection in 25% sucrose. 6 series of coronal sections of the brain of 30 μm of thickness were cut at the microtome and successively stored in an anti-freeze solution at -20°C until further processing. Free-floating brain sections were incubated overnight at room temperature with the following primaries antibodies: 1:500 anti-huntingtin (sc8767, Santa Cruz) and 1:2000 anti-p62 (GP62-C, Progen). The sections were then incubated with 1:200 biotinylated secondary antibody (horse anti-goat or goat anti guinea-pig, Vector Laboratories) followed by an avidin-biotin peroxidase solution (ABC Elite, Vector Laboratories). The staining was visualized with 3,39- diaminobenzidine (DAB).
