# Supplementary material for: Maintenance of Basal Levels of Autophagy in Huntington’s Disease Mouse Models Displaying Metabolic Dysfunction
Source: PLoS One. 2013 Dec 20;8(12):e83050. doi: 10.1371/journal.pone.0083050 (PMC3869748; doi:10.1371/journal.pone.0083050)
Supplement: Statistical Results S1 — (DOCX) [file pone.0083050.s007.docx]

**Statistical results S1**

Statistical significance is considered when p-values <0.05. For *post hoc* tests only p-values <0.05 are reported.

**Figure 1:**

A. Body weight for mice injected with rAAV5-htt853-18Q (18Q), rAAV5-htt853-79Q (79Q) and uninjected controls (C)

2-factor ANOVA:

Genotype: F_(2,195)_=115.018, p<0.001

Time: F_(4,195)_= 71.911, p<0.001

Genotype x Age: F_(8,195)_=14.098, p<0.001

1-factor ANOVA:

week 0: n.s. (F_(2,39)_=0.889)

week 2: F_(2,39)_=8.080, p=0.001

week 4: F_(2,39)_=28.812, p<0.001

week 6: F_(2,39)_=34.784, p<0.001

week 8: F_(2,39)_=47.785, p<0.001

Bonferroni post-hoc test:

week 2: 79Q vs 18Q p=0.02, 79Q vs C p=0.009

week 4: 79Q vs 18Q p<0.001, 79Q vs C p<0.001

week 6: 79Q vs 18Q p<0.001, 18Q vs C p=0.009, 79Q vs C p<0.001

week 8: 79Q vs 18Q p<0.001, 18Q vs C p=0.002, 79Q vs C p<0.001

**Figure 2:**

Expression of autophagy markers in the hypothalamus of rAAV5-htt853-18Q (18Q), rAAV5-htt853-79Q (79Q) and uninjected controls (C) measured by Western blot

1-factor ANOVA:

LC3II/I: n.s. (F_(2,17)_=0.018)

Atg7: n.s. (F_(2,17)_=0.08)

Beclin1: n.s. (F_(2,17)_=0.380)

p62: n.s. (F_(2,17)_=0.610)

pS6: n.s. (F_(2,17)_=0.284)

**Figure 3:**

Expression of autophagy markers in the hypothalamus of BACHD and wild-type mice at 2, 6 and 12 months of age measured by Western blot

LC3II/I:

2-factor ANOVA:

Genotype: n.s. (F_(1,21)_=0.150)

Age: n.s. (F_(2,21)_=1.214)

Genotype x Age: n.s. (F_(2,21)_=0.221)

Atg7:

2-factor ANOVA:

Genotype: n.s. (F_(1,21)_=0.328)

Age: n.s. (F_(2,21)_=0.859)

Genotype x Age: n.s. (F_(2,21)_=1.320)

Beclin1:

2-factor ANOVA:

Genotype: n.s. (F_(1,21)_=0.567)

Age: n.s. (F_(2,21)_=0.250)

Genotype x Age: n.s. (F_(2,21)_=1.936)

p62:

2-factor ANOVA:

Genotype: n.s. (F_(1,20)_=1.188)

Age: F_(2,20)_=16.278, p<0.001

Genotype x Age: n.s. (F_(2,20)_=0.493)

1-factor ANOVA:

wt: F_(2,10)_=10.115, p=0.004

BACHD: F_(2,10)_=7.134, p=0.012

Bonferroni post-hoc test:

wt: 2mo vs 12mo p=0.003

BACHD: 2mo vs 12mo p=0.02

**Figure 4:**

Expression of autophagy markers in the cerebral cortex of BACHD and wild-type mice at 2, 6 and 12 months of age measured by Western blot

LC3II/I:

2-factor ANOVA:

Genotype: F_(1,25)_=4.715, p=0.040

Age: n.s. (F_(2,25)_=1.871)

Genotype x Age: n.s. (F_(2,25)_=0.990)

Student’s un-paired T-test:

At 12mo p=0.006

Atg7:

2-factor ANOVA:

Genotype: n.s. (F_(1,25)_=0.020)

Age: n.s. (F_(2,25)_=0.044)

Genotype x Age: n.s. (F_(2,25)_=1.919)

Beclin1:

2-factor ANOVA:

Genotype: n.s. (F_(1,25)_=0.003)

Age: n.s. (F_(2,25)_=0.165)

Genotype x Age: n.s. (F_(2,25)_=0.625)

p62:

2-factor ANOVA:

Genotype: n.s. (F_(1,24)_=1.150)

Age: F_(2,24)_=8.329, p=0.002

Genotype x Age: n.s. (F_(2,24)_=0.102)

1-factor ANOVA:

wt: n.s. F_(2,11)_=2.961

BACHD: F_(2,13)_=5.941, p=0.015

Bonferroni post-hoc test:

BACHD: 2mo vs 12mo p=0.013

**Figure 5:**

Expression of autophagy markers in the striatum of BACHD and wild-type mice at 2, 6 and 12 months of age measured by Western blot

LC3II/I:

2-factor ANOVA:

Genotype: n.s. (F_(1,25)_=3.514)

Age: n.s. (F_(2,25)_=1.975)

Genotype x Age: n.s. (F_(2,25)_=0.098)

Atg7:

2-factor ANOVA:

Genotype: n.s. (F_(1,25)_=0.364)

Age: n.s. (F_(2,25)_=0.228)

Genotype x Age: n.s. (F_(2,25)_=0.310)

Beclin1:

2-factor ANOVA:

Genotype: n.s. (F_(1,25)_=2.211)

Age: n.s. (F_(2,25)_=0.023)

Genotype x Age: n.s. (F_(2,25)_=0.217)

p62:

2-factor ANOVA:

Genotype: n.s. (F_(1,25)_=1.441)

Age: n.s. (F_(2,25)_=1.839)

Genotype x Age: n.s. (F_(2,25)_=0.384)

**Table 2:**

Expression of autophagy markers in the hypothalamus of rAAV5-htt853-18Q (18Q), rAAV5-htt853-79Q (79Q) and uninjected controls (C) measured by Q-RT-PCR

1-factor ANOVA:

LC3B: n.s. (F_(2,18)_=3.450)

LC3A: n.s. (F_(2,18)_=1.058)

Atg5: n.s. (F_(2,18)_=1.139)

Atg7: n.s. (F_(2,18)_=0.498)

Beclin1: n.s. (F_(2,18)_=1.120)

p62: n.s. (F_(2,18)_=0.549)

LAMP2: n.s. (F_(2,18)_=1.047)

mTor: n.s. (F_(2,18)_=2.841)

Raptor: n.s. (F_(2,18)_=2.038)

Rictor: n.s. (F_(2,18)_=0.395)

**Table 3:**

Expression of autophagy markers in the hypothalamus of BACHD and wild-type mice at 2, 6 and 12 months of age measured by Q-RT-PCR

LC3B:

2-factor ANOVA:

Genotype: F_(1,21)_=6.451, p=0.019

Age: n.s. (F_(2,21)_=1.518)

Genotype x Age: n.s. (F_(2,21)_=0.168)

Student’s un-paired T-test:

At 2mo: n.s. p= 0.412

At 6mo: n.s. p= 0.089

At 12mo: n.s. p= 0.127

LC3A:

2-factor ANOVA:

Genotype: n.s. (F_(1,21)_=0.707)

Age: F_(2,21)_=3.875, p=0.037

Genotype x Age: n.s. (F_(2,21)_=0.781)

1-factor ANOVA:

wt: n.s. (F_(2,10)_=3.492)

BACHD: n.s. (F_(2,13)_=1.079)

Atg5:

2-factor ANOVA:

Genotype: F_(1,21)_=6.047, p=0.023

Age: n.s. (F_(2,21)_=1.351)

Genotype x Age: n.s. (F_(2,21)_=0.736)

Student’s un-paired T-test:

At 2mo: n.s. p= 0.727

At 6mo: n.s. p= 0.095

At 12mo: p= 0.044

Atg7:

2-factor ANOVA:

Genotype: n.s. (F_(1,21)_=0.059)

Age: n.s. (F_(2,21)_=1.485)

Genotype x Age: n.s. (F_(2,21)_=1.391)

Beclin1:

2-factor ANOVA:

Genotype: n.s. (F_(1,21)_=0.228)

Age: n.s. (F_(2,21)_=0.183)

Genotype x Age: n.s. (F_(2,21)_=0.961)

p62:

2-factor ANOVA:

Genotype: n.s. (F_(1,21)_=0.055)

Age: F_(2,21)_=6.848, p=0.005

Genotype x Age: n.s. (F_(2,21)_=1.534)

1-factor ANOVA:

wt: F_(2,10)_=4.302, p=0.045

BACHD: F_(2,13)_=4.005, p=0.049

Bonferroni post-hoc test:

wt: 2mo vs 6mo p=0.048

LAMP2:

2-factor ANOVA:

Genotype: F_(1,21)_=5.704, p=0.026

Age: n.s. (F_(2,21)_=1.224)

Genotype x Age: n.s. (F_(2,21)_=0.850)

Student’s un-paired T-test:

At 2mo: n.s. p= 0.192

At 6mo: n.s. p= 0.101

At 12mo: n.s. p= 0.587

mTor:

2-factor ANOVA:

Genotype: n.s. (F_(1,21)_=2.211)

Age: n.s. (F_(2,21)_=0.266)

Genotype x Age: n.s. (F_(2,21)_=1.000)

Raptor:

2-factor ANOVA:

Genotype: n.s. (F_(1,21)_=3.488)

Age: n.s. (F_(2,21)_=0.644)

Genotype x Age: n.s. (F_(2,21)_=0.493)

Rictor:

2-factor ANOVA:

Genotype: n.s. (F_(1,21)_=0.051)

Age: n.s. (F_(2,21)_=0.222)

Genotype x Age: n.s. (F_(2,21)_=1.852)

**Table 4:**

Expression of autophagy markers in the cerebral cortex of BACHD and wild-type mice at 2, 6 and 12 months of age measured by Q-RT-PCR

LC3B:

2-factor ANOVA:

Genotype: n.s. (F_(1,25)_=4.044)

Age: n.s. (F_(2,25)_=0.816)

Genotype x Age: n.s. (F_(2,25)_=0.199)

LC3A:

2-factor ANOVA:

Genotype: n.s. (F_(1,25)_=1.300)

Age: n.s. (F_(2,25)_=1.312)

Genotype x Age: n.s. (F_(2,25)_=1.329)

Atg5:

2-factor ANOVA:

Genotype: n.s. (F_(1,25)_=0.001)

Age: n.s. (F_(2,25)_=0.033)

Genotype x Age: n.s. (F_(2,25)_=0.534)

Atg7:

2-factor ANOVA:

Genotype: F_(1,25)_=14.233, p=0.001

Age: n.s. (F_(2,25)_=0.084)

Genotype x Age: n.s. (F_(2,25)_=1.872)

Student’s un-paired T-test:

At 2mo: n.s. p= 0.234

At 6mo: n.s. p= 0.126

At 12mo: p= 0.006

Beclin1:

2-factor ANOVA:

Genotype: n.s. (F_(1,25)_=1.523)

Age: F_(2,25)_=3.900, p=0.034

Genotype x Age: n.s. (F_(2,25)_=0.730)

1-factor ANOVA:

wt: F_(2,12)_=5.448, p=0.021

BACHD: n.s. (F_(2,13)_=0.655)

Bonferroni post-hoc test:

wt: 2mo vs 12mo p=0.034

p62:

2-factor ANOVA:

Genotype: n.s. (F_(1,25)_=2.291)

Age: n.s. (F_(2,25)_=0.252)

Genotype x Age: n.s. (F_(2,25)_=1.217)

LAMP2:

2-factor ANOVA:

Genotype: (F_(1,25)_=1.872)

Age: n.s. (F_(2,25)_=0.681)

Genotype x Age: n.s. (F_(2,25)_=1.418)

mTor:

2-factor ANOVA:

Genotype: n.s. (F_(1,25)_=0.116)

Age: n.s. (F_(2,25)_=0.466)

Genotype x Age: n.s. (F_(2,25)_=2.129)

Raptor:

2-factor ANOVA:

Genotype: n.s. (F_(1,25)_=0.546)

Age: n.s. (F_(2,25)_=1.683)

Genotype x Age: n.s. (F_(2,25)_=1.444)

Rictor:

2-factor ANOVA:

Genotype: n.s. (F_(1,25)_=0.886)

Age: n.s. (F_(2,25)_=0.712)

Genotype x Age: n.s. (F_(2,25)_=0.811)

**Table 5:**

Expression of autophagy markers in the striatum of BACHD and wild-type mice at 2, 6 and 12 months of age measured by Q-RT-PCR

LC3B:

2-factor ANOVA:

Genotype: n.s. (F_(1,25)_=.392)

Age: n.s. (F_(2,25)_=0.867)

Genotype x Age: n.s. (F_(2,25)_=0.529)

LC3A:

2-factor ANOVA:

Genotype: n.s. (F_(1,25)_=3.200)

Age: n.s. (F_(2,25)_=0.187)

Genotype x Age: n.s. (F_(2,25)_=0.612)

Atg5:

2-factor ANOVA:

Genotype: n.s. (F_(1,25)_=3.644)

Age: n.s. (F_(2,25)_=0.029)

Genotype x Age: n.s. (F_(2,25)_=0.022)

Atg7:

2-factor ANOVA:

Genotype: n.s. (F_(1,25)_=0.123)

Age: n.s. (F_(2,25)_=1.753)

Genotype x Age: n.s. (F_(2,25)_=0.024)

Beclin1:

2-factor ANOVA:

Genotype: n.s. (F_(1,25)_=0.316)

Age: n.s. (F_(2,25)_=1.366)

Genotype x Age: n.s. (F_(2,25)_=0.235)

p62:

2-factor ANOVA:

Genotype: F_(1,25)_=17.301, p<0.001

Age: n.s. (F_(2,25)_=0.517)

Genotype x Age: n.s. (F_(2,25)_=2.246)

Student’s un-paired T-test:

At 2mo: n.s. p= 0.252

At 6mo: p= 0.018

At 12mo: p= 0.010

LAMP2:

2-factor ANOVA:

Genotype: (F_(1,25)_=1.504)

Age: n.s. (F_(2,25)_=0.393)

Genotype x Age: n.s. (F_(2,25)_=0.611)

mTor:

2-factor ANOVA:

Genotype: n.s. (F_(1,25)_=0.006)

Age: n.s. (F_(2,25)_=0.615)

Genotype x Age: n.s. (F_(2,25)_=0.831)

Raptor:

2-factor ANOVA:

Genotype: n.s. (F_(1,25)_=0.240)

Age: n.s. (F_(2,25)_=0.096)

Genotype x Age: n.s. (F_(2,25)_=0.193)

Rictor:

2-factor ANOVA:

Genotype: n.s. (F_(1,25)_=0.197)

Age: n.s. (F_(2,25)_=0.543)

Genotype x Age: n.s. (F_(2,25)_=0.672)
